# Comments on “Neoadjuvant hepatic arterial infusion of oxaliplatin, fluorouracil, and leucovorin for resectable single large hepatocellular carcinoma”

**DOI:** 10.1097/JS9.0000000000003267

**Published:** 2025-08-26

**Authors:** Jiale Li, Ying Xia, Zigui Chen

**Affiliations:** Department of Neurosurgery, Haikou Affiliated Hospital of Central South University Xiangya School of Medicine, Haikou, China

*Dear Editor,*

We read with great interest the recent study titled “Neoadjuvant hepatic arterial infusion of oxaliplatin, fluorouracil, and leucovorin for resectable single large hepatocellular carcinoma,” which explores whether neoadjuvant hepatic arterial infusion chemotherapy (HAIC) can improve surgical outcomes for patients with resectable single large hepatocellular carcinoma (HCC)^[1]^. While this research adds valuable insights, several critical methodological, statistical, and interpretative issues require further clarification and improvement. In accordance with the TITAN guideline, we confirm that artificial intelligence was used solely for language polishing and not for content generation. The authors assume full responsibility for the integrity of the final manuscript^[2]^.

First, the study uses the initial diagnosis date as the starting point for survival analysis, introducing potential immortal-time bias, as the neoadjuvant group inherently experiences a guaranteed survival period while receiving treatment. Landmark analysis starting from the date of surgery or other appropriate time-dependent statistical methods could mitigate this bias and yield more accurate estimates of treatment efficacy.

Second, the retrospective nature of this multi-center study and the use of propensity score matching (PSM) may leave residual confounding. Although PSM attempts to balance baseline characteristics, critical variables such as portal hypertension indicators, body mass index (BMI), antiviral therapy, and nutritional or inflammatory status were not accounted for, potentially skewing results.

Third, the matching procedure itself employed a relatively broad caliper (0.20), which might allow significant residual imbalance between groups, compromising the internal validity of the findings. A more stringent matching or adjustment through inverse probability treatment weighting (IPTW) may enhance the robustness of the results.

Fourth, safety and toxicity profiles require deeper exploration. The study reports increased postoperative bile leaks and transient elevations in transaminases associated with HAIC. However, late-onset adverse events such as neurotoxicity, cumulative myelosuppression, or delayed sinusoidal obstruction syndrome were not systematically evaluated or reported. More comprehensive toxicity assessment is necessary to fully characterize treatment safety.

Fifth, the reduction in microvascular invasion (MVI) after HAIC may reflect selection bias, as patients responding well to chemotherapy likely represent a subgroup with favorable tumor biology. Including MVI simultaneously in multivariate survival analyses alongside HAIC treatment could inadvertently introduce mediator bias, underestimating the independent effect of HAIC.

Finally, clinical management heterogeneity post-surgery, such as additional adjuvant therapy or antiviral treatments, was not clearly delineated, possibly confounding long-term outcomes. Detailed reporting of postoperative treatments and adjustments for potential confounding are essential for accurate interpretation of long-term survival and disease-free survival outcomes.

In summary, this commentary provides a critical appraisal of a recent study on neoadjuvant HAIC for resectable large hepatocellular carcinoma, addressing methodological and interpretative issues that may affect the validity of its conclusions. By identifying potential immortal-time bias, residual confounding due to incomplete covariate adjustment, limitations in matching procedures, inadequate evaluation of long-term toxicities, and the risk of mediator bias in multivariate analyses, this article adds clarity to ongoing debates regarding the clinical utility of HAIC in this context. These insights highlight the importance of rigorous trial design, comprehensive safety assessment, and careful statistical handling in future studies, offering valuable learning points for clinicians, researchers, and methodologists involved in hepatocellular carcinoma management.

## Data Availability

None.

## References

[1] Hu Z Deng M Fu Y. Neoadjuvant hepatic arterial infusion of oxaliplatin, fluorouracil, and leucovorin for resectable single large hepatocellular carcinoma. Int J Surg 2025;111:3850–58. 40358645 10.1097/JS9.0000000000002437 PMC12165535

[2] Agha RA Mathew G Rashid R. Transparency In The reporting of Artificial INtelligence – the TITAN guideline. Prem J Sci 2025;10:100082.

